# Insights into *Pasteurellaceae* carriage dynamics in the nasal passages of healthy beef calves

**DOI:** 10.1038/s41598-019-48007-5

**Published:** 2019-08-16

**Authors:** A. C. Thomas, M. Bailey, M. R. F. Lee, A. Mead, B. Morales-Aza, R. Reynolds, B. Vipond, A. Finn, M. C. Eisler

**Affiliations:** 1 Bristol Veterinary School, University of Bristol, Langford, UK; 2 Rothamsted Research, North Wyke, Devon, UK; 3 Rothamsted Research, Harpenden, UK; 4 Bristol Children’s Vaccine Centre, University of Bristol, Bristol, UK; 5 School of Cellular and Molecular Medicine, University of Bristol, Bristol, UK; 6 School of Population Health Sciences, University of Bristol, Bristol, UK; 7 Public Health Laboratory Bristol, Public Health England, Bristol, UK

**Keywords:** Infectious-disease epidemiology, Bacteriology

## Abstract

We investigated three bovine respiratory pathobionts in healthy cattle using qPCR optimised and validated to quantify *Histophilus somni*, *Mannheimia haemolytica* and *Pasteurella multocida* over a wide dynamic range. A longitudinal study was conducted to investigate the carriage and density of these bacteria in the nasal passages of healthy beef calves (N = 60) housed over winter in an experimental farm setting. The three pathobiont species exhibited remarkably different carriage rates and density profiles. At housing, high carriage rates were observed for *P*. *multocida* (95%), and *H*. *somni* (75%), while fewer calves were positive for *M*. *haemolytica* (13%). Carriage rates for all three bacterial species declined over the 75-day study, but not all individuals became colonised despite sharing of environment and airspace. Colonisation patterns ranged from continuous to intermittent and were different among pathobiont species. Interval-censored exponential survival models estimated the median duration of *H*. *somni* and *P*. *multocida* carriage at 14.8 (CI_95%_: 10.6–20.9) and 55.5 (CI_95%_: 43.3–71.3) days respectively, and found higher density *P*. *multocida* carriage was associated with slower clearance (p = 0.036). This work offers insights into the dynamics of pathobiont carriage and provides a potential platform for further data collection and modelling studies.

## Introduction

Respiratory microbiota are diverse communities in humans and livestock^1^. In humans, *Staphylococcus aureus* and *Streptococcus pneumoniae* are frequently carried in the nasal passages as commensals but are also significant pathogens on occasion^2^. Bacteria with this dual commensal-pathogen behaviour have been dubbed pathobionts, although key drivers between commensal and pathogenic states remain to be identified.

Similarly, healthy cattle carry pathobionts in their nasal passages, most commonly members of the *Pasteurellaceae* family: *Histophilus somni*, *Mannheimia haemolytica* and *Pasteurella multocida*^3,4^. In the UK, these bacteria are frequently identified in clinical cases of bovine respiratory disease (BRD), along with *Mycoplasma bovis* and viruses including bovine respiratory syncytial virus (BRSV), parainfluenza virus-3 (BPI3-V), bovine herpesvirus-1 (BHV-1) and bovine diarrhoea virus (BVDV)^5^. Although the relative importance for disease causation of these various microbial species and their capacity to transit from harmless colonisation of the upper respiratory tract to harmful infection of the lower respiratory tract certainly varies, transition may also be associated with possession of specific virulence factors such as leukotoxin in *M*. *haemolytica*^6–8^.

*Pasteurellaceae* carriage appears to be benign without predisposing factors such as respiratory viral infection or environmental stressors (e.g. temperature, humidity, transport, mixing of groups of animals); these are thought to be associated with bacterial proliferation and migration into the lower respiratory tract, resulting in pneumonia^9–11^. Respiratory disease can affect cattle of any age, but is most commonly seen in calves aged under 6 months, typically while housed or shortly after transportation, hence the clinical terminology ‘enzootic pneumonia’ or ‘shipping fever’^12^. Significant economic losses are incurred, resulting from decreased production, increased morbidity and mortality, and through increased costs of husbandry, veterinary care and preventive measures^13^. Control of BRD is directed at improving management conditions and preventing viral and bacterial infections through vaccination and antimicrobial therapy^14,15^. However, reports of resistance to antimicrobials including newer compounds are common and of increasing concern^16–18^.

Bacterial carriage in the bovine upper respiratory tract has generally been detected by culture^19–23^, but this is problematic due to the fastidious nature of the organisms and overgrowth by faster-growing species^24^. Advances in molecular techniques have allowed in-depth investigation of organisms that are currently difficult or impossible to culture, for example by PCR^25^ and sequencing^3,26,27^. Real-time quantitative PCR (qPCR) is a rapid and specific method to reliably detect and measure density of bacterial species in a range of clinical samples. TaqMan qPCR uses a 5′ hydrolysis probe alongside oligonucleotide primers to enhance assay specificity^28^. Density of carriage of bovine respiratory pathobionts has not previously been investigated using qPCR to determine organism load, which may be an important predictor or determinant of disease.

Bacterial carriage is often assessed over time by repeated sampling and assaying for the presence or absence of target organisms. Inherently, this longitudinal approach gives rise to interval-censored data, when an event of interest is known to occur within an interval^29^. In humans, upper respiratory tract pneumococcal carriage has been modelled using exponential interval-censored survival models to determine the rate of clearance and duration of carriage^30^, but these approaches are rarely used in veterinary research^29^.

In the studies reported here, we optimised three TaqMan qPCR assays for detection and quantification of *Histophilus somni*, *Mannheimia haemolytica* and *Pasteurella multocida* in bovine nasal swabs, and applied these assays to the investigation of rates and densities of carriage in the nasal passages of healthy beef calves. Using interval-censored exponential survival analyses, we modelled the time to bacterial clearance and investigated whether sex and density of carriage influenced the duration of nasal bacterial carriage. This approach offers novel insights into *Pasteurellaceae* carriage patterns in healthy livestock. Characterising bacterial colonisation dynamics in healthy animals should improve our understanding of the biology of carriage, including transmission dynamics, and in turn help inform prevention and control strategies.

## Results

### Growth curves in liquid culture

Pure cultures were maintained throughout growth curve studies for each bacterial species (*H*. *somni*, *M*. *haemolytica* and *P*. *multocida*), as confirmed by well-isolated colonies and pure Gram stain of overnight plate cultures. Ten-fold serial dilutions yielded appropriate colony counts for all three bacterial species. Growth rates of *H*. *somni*, *M*. *haemolytica* and *P*. *multocida* determined by optical density measurements can be seen in Supplementary Fig. S1.

### Real-time PCR standard curves and assay performance

Cultures of *H*. *somni*, *M*. *haemolytica* and *P*. *multocida* in liquid medium were used to construct standard curves for each organism (values given in Table 1 and shown in Supplementary Fig. S2) using the same PCR conditions used to amplify DNA targets in nasal swabs stored in skim milk-tryptone-glucose-glycerol (STGG). Linear regression of C_q_ value versus observed and extrapolated log_10_ mean colony count/ml for corresponding 10-fold serial dilutions of bacterial broth cultures provided equations for conversion of C_q_ values to CFU/ml^31^. These equations were later applied to C_q_ values obtained from nasal swabs to obtain genome copies/ml. For all standard curve dilutions, bacteriophage T4 internal amplification controls gave C_q_ values as expected, indicating successful DNA extraction and providing no evidence of PCR inhibition.

**Table 1:** Evaluation of *Histophilus somni*, *Mannheimia haemolytica* and *Pasteurella multocida* qPCR assays on pure log-phase broth cultures of reference strains.

Assay characteristic		*Histophilus somni*	*Mannheimia haemolytica*	*Pasteurella multocida*
Standard Equation	Slope (standard error)	−0.295 (0.00610)	−0.292 (0.00688)	−0.264 (0.00332)
	Intercept	11.3	12.4	11.2
	r^2^	0.995	0.991	0.998
	Biological replicates	2	3	3
	Degrees of freedom (total)	13	17	14
Efficiency (%)^†^		97.5	96.0	83.5
Linear dynamic range (log_10_)		8	7	6
C_q_ cut-off value^‡^		35 cycles	34 cycles	35 cycles

Linear regression of log_10_ colony count/ml against cycle quantification (C_q_) value to generate standard equation for conversion of swab C_q_ value to genome copies/ml.

^†^PCR Amplification Efficiency (%) = [(10^−slope^) − 1] × 100.

^‡^The C_q_ value corresponding to the endpoint dilution of the standard curve at which samples tested positive.

All assays were highly reproducible and repeatable for all bacterial species, producing consistent C_q_ values at each dilution of the standard curve for biological replicates tested on separate days (coefficient of variation, CV < 6%), and for technical replicates performed on the same day of testing (CV < 4%). For all standard curves, coefficient of determination values were high (r^2^ > 0.99). Amplification efficiency was > 95% for both *M*. *haemolytica* and *H*. *somni* and lower for *P*. *multocida* at 84% (Table 1). For all three assays, DNA extracts from three dilutions of all heterologous bacterial strains comprising a specificity panel (Supplementary Table S1) yielded negative results.

### Re-confirmation of PCR results by culture and sequencing

All nasal swabs collected at day 0 (N = 60) were cultured for the presence of *H*. *somni*, *M*. *haemolytica* and *P*. *multocida*. Presumptive and phenotypically confirmed isolates were stored at −70 °C. As only one swab was determined positive for *H*. *somni* by culture, PCR products from *H*. *somni* PCR positive nasal swabs (N = 5) were sequenced to confirm identity. In all five, the sequences were representative of *H*. *somni* as evident following Basic Local Alignment Search Tool (BLAST) searches: 100% homology to *H*. *somni* was seen for 3 of 5 queried sequences, 99% to the fourth and 98% to the fifth. For all 5 queried swab sequences there was homology between 96 and 100% for sequences deposited as ‘uncultured bacterium clone’, probably representing *H*. *somni*. One swab had 93% homology to one sequence deposited as *Actinobacillus capsulatus*. The majority of these swabs (43/60) were determined positive for *P*. *multocida* by both culture and PCR, with a smaller number PCR positive and culture negative (14/60). Fewer swabs were determined positive for *M*. *haemolytica* (4/60) by both culture and PCR. Similarly, a number (5/60) were PCR positive and culture negative. No swabs were culture positive but negative in the PCR for corresponding species (Supplementary Table S2).

### Characteristics of the study animals and signs of respiratory disease

Animal characteristics (sex, sire breed and age) are shown in Table 2. Animals were monitored for signs of respiratory disease using the Wisconsin calf respiratory scoring system. Nasal discharges were observed on three occasions (scored between 1 and 3), two of which were in the same animal. Coughing was observed in one animal on one occasion. No *Dictyocaulus viviparous* larvae were detected in faeces of any animal.

**Table 2:** Sex, sire breed and age of calves in the study (N = 60).

	Green Barn (N = 30)	Red Barn (N = 30)	Total
**Sex**			
Heifer	15/30	14/30	29/60
Steer	15/30	16/30	31/60
**Sire Breed**			
Charolais	20/30	21/30	41/60
Hereford	4/30	3/30	7/60
Limousin	6/30	6/30	12/60
**Median Age (day 0)**			
Days (range)	305 (276–319)	307 (258–324)	305 (258–324)

### Carriage rates and patterns

A total of 299 nasal swabs obtained during winter housing of calves were analysed by qPCR to determine carriage rates and densities of *H*. *somni*, *M*. *haemolytica* and *P*. *multocida*; 238 swabs were positive for at least one of these three target bacteria. *P*. *multocida* was most frequently detected (227/299; 75.9%), followed by *H*. *somni* (80/299; 26.8%) and *M*. *haemolytica* (17/299; 5.7%). The overall proportions of swabs positive in each barn were similar for *P*. *multocida* (Green Barn: 118/150; 78.7%, Red Barn: 109/149; 73.2%; p = 0.327) and *M*. *haemolytica* (Green Barn: 7/150; 4.7%, Red Barn: 10/149; 6.7%; p = 0.608). The proportion of swabs positive for *H*. *somni* was lower in the Green (20/150; 13.3%) than in the Red Barn (60/149; 40.3%; p < 0.001). In all cases, the T4 internal amplification control assay C_q_ values were as expected, indicating successful DNA extraction with no evidence of PCR inhibition.

**Figure 1:**
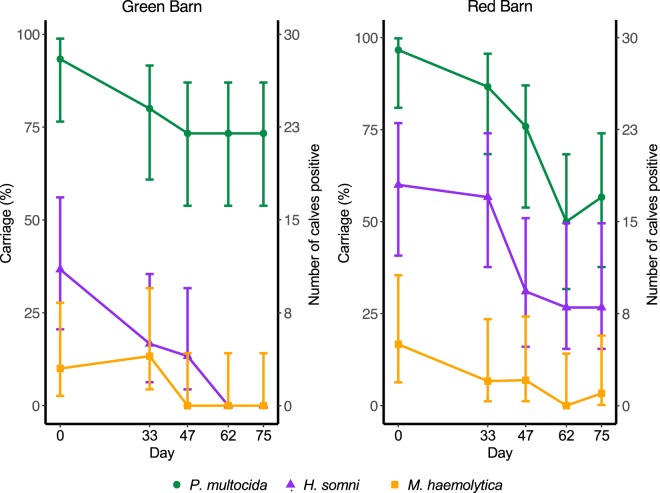
Nasal carriage of *Pasteurella multocida* (green circles), *Histophilus somni* (purple triangles) and *Mannheimia haemolytica* (orange squares) in healthy beef calves determined by qPCR on nasal swabs collected on five occasions from sixty calves housed in two identical barns (N = 30 Green Barn, N = 30 Red Barn). Error bars represent Newcombe 95% confidence intervals for the single proportion.

Target bacterial nucleic acid was detected in swabs from each of the sixty animals on at least one sampling occasion. Carriage rates were highest on the first occasion of sampling (day 0), and declined for all bacterial species thereafter, with similar trends observed in both barns. *P*. *multocida* was carried at a higher rate than *H*. *somni* and *M*. *haemolytica* on all sampling occasions in the Green Barn, and on days 0 and 47 in the Red Barn. Clearance of *H*. *somni* and *M*. *haemolytica* was observed in the Green barn from days 62 and 47, respectively (Fig. 1). Log-linear models provided no evidence to suggest any difference in carriage rates between barns and pens (see Supplementary Information, Method and Data S1).

**Figure 2:**
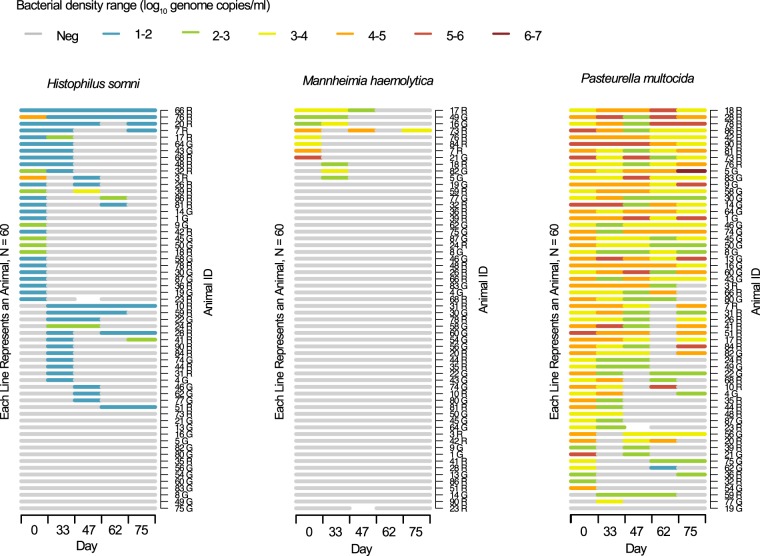
Carriage patterns and density of *Histophilus somni*, *Mannheimia haemolytica* and *Pasteurella multocida* determined by qPCR on nasal swabs collected on five sampling days (0, 33, 47, 62 and 75) from sixty calves. Animals housed in Green and Red barns are identified by G and R following ID numbers respectively. Density is represented as log_10_ genome copies/ml. One animal (ID 23 R) had a missing sample at day 47.

A carriage episode was defined as a period of carriage detected in consecutive samples without interruption. Forty-five (75%) of the 60 calves were positive for *H*. *somni* on at least one occasion: 36 of these calves had a single carriage episode and 9 had two episodes. *H*. *somni* was detected at the first visit in 29 calves, of which two remained positive at all subsequent study visits. Details of individual carriage trajectories are shown in Fig. 2.

Carriage of *P*. *multocida* was detected in 57 (95%) of the 60 calves at day 0: 38 of these calves had a single carriage episode and 19 had two episodes. Carriage was detected in 26 calves on all study visits. Of three calves negative for *P*. *multocida* on day 0, carriage was detected in two at the subsequent visit, while the third remained negative throughout the study.

Carriage of *M*. *haemolytica* was detected in 8 (13%) of the 60 animals at the first visit, of which 7 had one carriage episode and one had three. In three further calves, *M*. *haemolytica* was detected at the second visit, while this species of pathobiont was not detected in the remaining 49 animals at any visit.

Co-carriage was defined as detection of more than one bacterial species in the same sample and co-carriage of two and three species was found in 47 and three animals respectively. Co-carriage with *H*. *somni* and *P*. *multocida* occurred most frequently and *M*. *haemolytica* was co-carried with *P*. *multocida* more commonly than with *H*. *somni*. No evidence of association between pairs of species was apparent using Fisher’s exact test (p > 0.221 for all nine pairwise comparisons; six pairwise comparisons were not performed because neither of the relevant pair of bacterial species were present; p-values may be underestimated as we have not controlled for multiple comparisons).

### Carriage density

**Figure 3:**
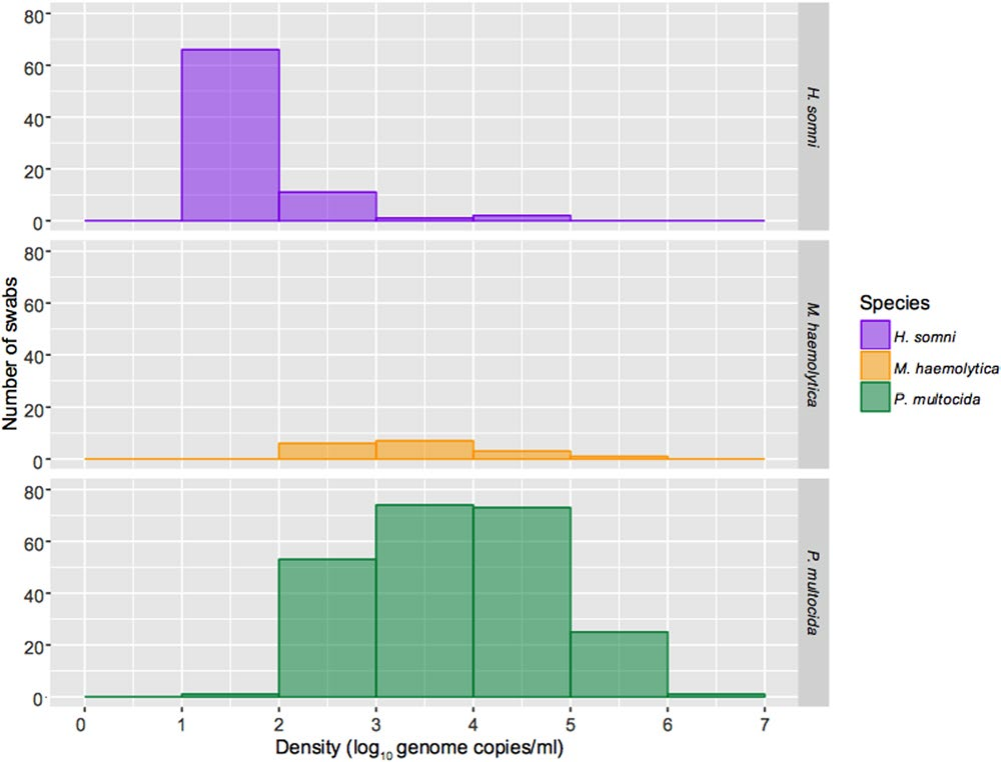
Histograms for all positive swabs summarising density distribution profiles of *Histophilus somni* (N = 80), *Mannheimia haemolytica* (N = 17) and *Pasteurella multocida* (N = 227). Note these data include up to 5 positive swabs from single animals, as shown in Fig. 2.

Individual animal density data are shown in Fig. 2. For each bacterium, density values for all positive samples were also plotted as histograms revealing distinct density profiles (Fig. 3). The modal value for *H*. *somni* carriage density accounting for the vast majority (82.5%) of swabs was 1–2 log_10_ genome copies/ml with fewer samples (13.8%) between 2 and 3 logs. Only two samples in two calves were in the 4–5 log range and one sample in the 3–4 log range. Although carriage rates were low for *M*. *haemolytica*, carriage density ranged between 2 and 6 logs when it occurred. *Pasteurella multocida* was also carried over a wide range of densities, most commonly between 3 and 5 log_10_ genome copies/ml, extending up to 6.08 (ID: 5 G) logs in one sample. Although density of *P*. *multocida* carriage was also dynamic within and between calves, some animals maintained carriage at high densities in the 4–6 log range over several sampling visits, and only one sample in one calf (ID: 62 G) was in the 1–2 log range (Fig. 2).

### Duration of carriage and hazard of clearance

**Figure 4:**
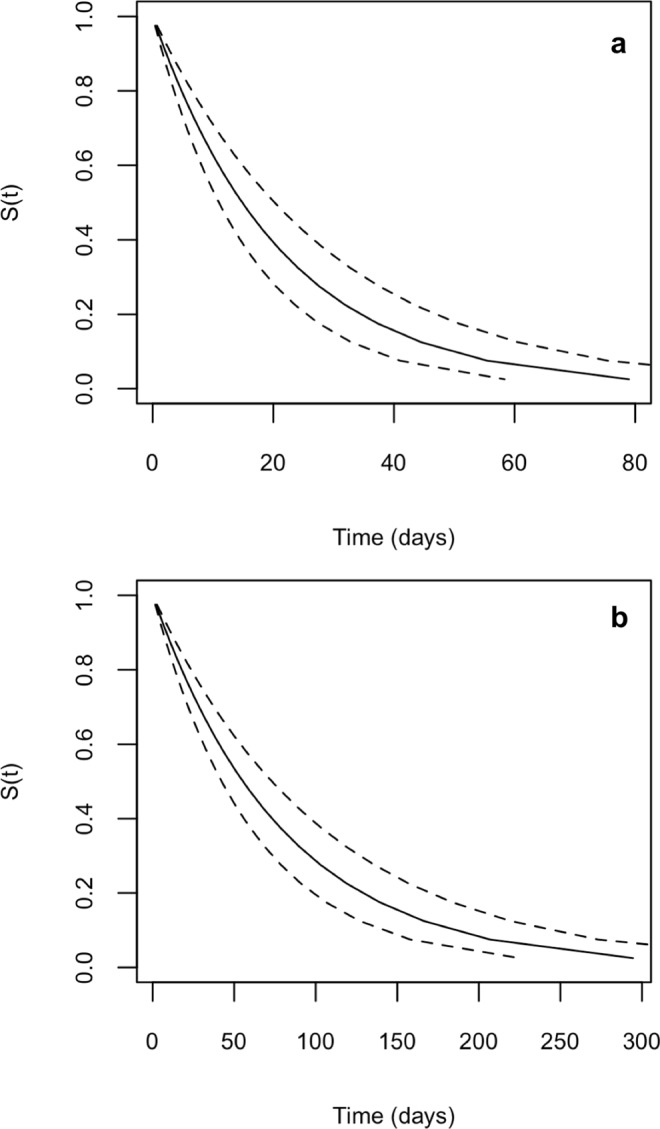
Survival curves: proportion of carriage episodes still ongoing by time for *Histophilus somni* (panel a) and *Pasteurella multocida* (panel b). Dotted lines represent 95% confidence interval.

Interval-censored exponential survival models for carriage (without inclusion of carriage density, host animal sex, barn or pen as covariates) estimated the median duration of *H*. *somni* carriage to be 14.8 days (CI_95%_: 10.6–20.9) and the hazard of clearance to be 0.0467 per day (CI_95%_: 0.0326–0.0634). For *P*. *multocida*, the median carriage duration was estimated to be 55.5 days (CI_95%_: 43.3–71.3) and hazard of clearance to be 0.0125 per day (CI_95%_: 0.00969–0.0159, Fig. 4). For either bacterium, the animals’ sex (*H*. *somni*: p = 0.414; *P*. *multocida*: p = 0.311), barn (*H*. *somni*: p = 0.060; *P*. *multocida*: p = 0.106) or pen (*H*. *somni*: 0.119; *P*. *multocida*: p = 0.449) did not significantly influence carriage duration in survival models. Carriage rates of *M*. *haemolytica* were too low for meaningful survival modelling. The effects of carriage density on carriage duration was modelled in univariable analyses. Log hazard ratio and density data were suggestive of a non-linear trend for both *H*. *somni* and *P*. *multocida*, therefore we modelled the effect of density using categories based on density quartiles rather than continuously. For *P*. *multocida*, density of carriage significantly influenced subsequent carriage duration (p = 0.036, Table 3). Categories 2–4 of *P*. *multocida* density were significantly associated with increased carriage duration compared to the reference category (1); these trends were non-linear with categories 2 and 4 associated with a longer duration (lower hazard) compared to category 3, and category 4 (highest density) associated with the longest subsequent carriage duration (Table 3). Density was not seen to affect *H*. *somni*.

**Table 3:** Interval-censored exponential survival models.

Species	Model	Covariate	Hazard ratio	CI_95%_ of hazard ratio	Hazard ratio p-value^§^	Log-likelihood
*H*. *somni* (N = 54)	M0: Unconditional		0.0467*	0.0333–0.0655		−55.8
	M1: Density^†^	Category 1 (N = 14)^1^	0.0556*	0.0261–0.112		−55.0
		Category 2 (N = 13)^2^	0.967	0.383–2.44	0.943	
		Category 3 (N = 13)^2^	0.610	0.219–1.69	0.342	
		Category 4 (N = 14)^2^	0.850	0.266–2.72	0.784	
	M2: Sex	Heifer^1^	0.0411*	0.0260–0.0617		−55.5
		Steer^2^	1.29	0.635–2.61	0.484	
*P*. *multocida* (N = 78)	M0: Unconditional		0.0125*	0.00950–0.0164		−109
	M1: Density^‡^	Category 1 (N = 20)^1^	0.0280*	0.0145–0.0524		−104
		Category 2 (N = 19)^2^	0.393	0.189–0.814	**0.0119**	
		Category 3 (N = 19)^2^	0.321	0.153–0.674	**0.00267**	
		Category 4 (N = 20)^2^	0.414	0.197–0.868	0.0196	
	M2: Sex	Heifer^1^	0.0108*	0.00791–0.0146		−108
		Steer^2^	1.33	0.464–1.23	0.255	

*H*. *somni* log-likelihood ratio test statistic: (M0 vs. M1), *χ*^2^ (3 df) = 1.62, p = 0.655; (Model 0 vs. Model 2), *χ*^2^ (1 df) = 0.668, p = 0.414.

*P*. *multocida* log-likelihood ratio test statistic: (M0 vs. M1), *χ*^2^ (3 df) = 8.52, p = 0.036; (Model 0 vs. Model 2), *χ*^2^ (1 df) = 1.028, p = 0.311.

*Hazard for unconditional model and baseline category.

^†^*H*. *somni* density categories based on quartiles (log_10_ genome copies/ml): <1.26; ≥1.26 to 1.45; ≥1.45 to 1.96; ≥1.96.

^‡^*P*. *multocida* density categories based on quartiles (log_10_ genome copies/ml): <3.40; ≥3.40 to 3.99; ≥3.99 to 4.77; ≥4.77.

^§^P-values shown are for the null hypothesis of no difference between each of the category levels^2^ in M1 (categories 2, 3 and 4) and M2 (steer) compared to their respective baseline categories^1^ (category 1 and heifer).

## Discussion

We present the first longitudinal investigation into nasal carriage patterns and densities of common bovine respiratory pathobionts in healthy animals using molecular detection tools. The detailed quantitative profiles we obtained over a typical housing period demonstrate marked differences between the bacteria studied in terms of frequency, duration of carriage and microbial density. These results provide methodological and biological information that will be important for future study of bacterial carriage and transmission in housed animals, and the impact of vaccines and viral infections upon respiratory disease.

PCR performed on nasal swabs may give a rapid and reliable indication of both the current colonisation status and, in the case of PCR-positive culture-negative samples, perhaps the recent footprint of colonisation as compared to conventional bacteriological culture which can only reflect the former. To validate detection of target bacteria using the qPCR assays presented in this study we cultured a subset of nasal swabs collected on day 0 (N = 60). In all cases, swabs negative by PCR were also negative by culture and detection of all species was enhanced using PCR, probably reflecting swabs with target DNA but too few viable organisms for successful culture. In addition, we experienced consistent difficulty in isolating *H*. *somni*. Selective agars (*Haemophilus* selective and chocolate with bacitracin) supported growth of type strain ATCC 43625 but no field isolates were obtained from nasal swabs by this method; instead *H*. *somni* was cultured from only one swab using non-selective chocolate agar. Reassuringly amplicons from five nasal swabs determined PCR positive (one of which was culture-positive) had sequence homology to *H*. *somni*. This highlights the difficulty with culture which requires specialist skills and can yield values for bacterial density only over a narrow range, unless very labour-intensive serial dilutions are prepared for each sample.

The *sodA* gene of *M*. *haemolytica* and 16S rRNA gene sequences of *H*. *somni* and *P*. *multocida* were proven to be qPCR primer targets capable of separating these pathobionts from other closely-related and common non-pathogenic bacterial species (Supplementary Table S1). Hitherto, discrimination between *M*. *haemolytica* and *M*. *glucosida* by PCR has generally relied either on analysis of melting curves following SYBR green qPCR, or on the assumption that PCR positive samples are *M*. *haemolytica* because *M*. *glucosida* has not been implicated in clinical cases of bovine respiratory disease^32,33^. We were able to discriminate successfully between *M*. *haemolytica* and all other members of the *Mannheimia* genus using a TaqMan probe. A well-known limitation of PCR is that it does not distinguish viable from non-viable organisms. We used cells harvested from liquid cultures in the exponential phase of growth to ensure that the bacterial cell populations for production of calibration curves were highly likely to be viable^31^.

Animals remained healthy and no animals were diagnosed with respiratory disease throughout the study; on only a few occasions were very slight nasal discharge or coughing observed, but these were mild and transient, and no treatment was necessary. No *Dictyocaulus viviparous* (bovine lung worm) larvae were detected on any occasion. Surveillance data on diagnostic submissions to Veterinary Investigation Centres confirm bacterial species involved in bovine pneumonia in the UK; most frequently *Mannheimia* spp. closely followed by *P*. *multocida*^5^. The prevalence of *P*. *multocida* as a commensal in the healthy bovine respiratory tract varies^21,24,34^; the high carriage rates by PCR (61.7–95.0%) we report here in healthy animals suggest it may function as part of the core nasal microbiota. By contrast, we rarely detected *M*. *haemolytica* (carriage rates of up to 13.3%), although when carriage did occur it was over a wide range of densities (2–6 logs). It has been suggested *M*. *haemolytica* is carried preferentially deeper in the nasopharynx at the palatine tonsil^35,36^, but our detection of this species in short nasal swabs suggests it may also be shed in nasal secretions. Using culture, nasal carriage of *H*. *somni* has been reported in healthy calves (6.6%) and those with respiratory disease (11.9%)^21^. We observed higher rates by PCR (13.3% to 48.3%). This may reflect the increased sensitivity of detection by qPCR compared to culture, suggesting that *H*. *somni* may have been underreported previously.

Patterns of carriage for all three pathobiont species were similar between barns but not identical (clearance of *H*. *somni* was observed in the Green barn only). This suggests epidemiology may vary somewhat in different but overtly similar groups of animals. Furthermore, carriage rates for all species declined over the study period in both barns. This observation may reflect animals becoming older. Older animals which are immunologically more mature, or those which have developed appropriate immune responses following exposure may be better equipped to clear bacteria than younger animals.

We defined an episode of carriage as a period when an animal was positive for any one bacterial species in consecutive samples without interruption. One animal experienced three such episodes of carriage with *M*. *haemolytica* and carriage was often transient for all three species. While such apparent transient carriage episodes may reflect genuine clearance and re-acquisition, negative results could also be due to intermediate and other sample(s) below the limit of detection of the assay, in the context of apparent large fluctuations in density over time (Fig. 2).

We do not know whether and to what extent the bacteria are evenly distributed within the upper respiratory tract of colonised animals. Conversely, apparent continuous episodes of carriage in our study could also represent repeated acquisition and successive carriage episodes with the same or different strains of *H*. *somni*, *M*. *haemolytica* or *P*. *multocida*. This is a limitation of repeated sampling studies which can be partially addressed by taking more frequent samples or undertaking more detailed characterisation of the bacteria detected. Approaches such as pulsed-field gel electrophoresis, multilocus sequence typing^37^ and whole genome sequencing can be used to study transmission events; however, their success is dependent on adequate genetic diversity^38^.

We did not find any evidence of interspecies competition between these three bacterial species which might have influenced their carriage rates. It is possible that carriage rates were too low for us to detect an association between bacterial species which has been reported by others^39,40^ with the number of animals in our study. Serotype displacement and replacement is known to occur following dysbiosis of the respiratory niche, for example pneumococcal carriage following universal vaccination in childhood^41^. In cattle, although differences in serotype prevalence have been suggested between healthy animals and those suffering with respiratory disease, this area of microbiology is relatively unexplored^42,43^.

Interval-censoring occurs when the time-to-event is not observed precisely but is known to have occurred within a particular interval. This commonly occurs in monitoring for infectious diseases when discrete time points are chosen to monitor carriage/infection status^29^. Biased estimation can result if this imprecision is ignored and the midpoint or right end point of the observed interval is taken as the exact event/failure time^44^. Using interval-censored exponential survival analysis we estimated the median duration of *H*. *somni* carriage at 14.8 days (hazard; 0.0467 per day) and at 55.5 days for *P*. *multocida* (hazard; 0.0125 per day), concordant with the increased prevalence observed for *P*. *multocida*. We found increasing *P*. *multocida* carriage density was significantly associated with increased carriage duration. One explanation for reduced carriage duration at lower densities could be the requirement of a certain number of bacteria for colonisation^45^, however at higher densities (over a certain threshold) the opportunity for naïve individuals to become exposed increases. Incidentally, *H*. *somni* was predominately carried at lower densities (1–2 logs) and was not significantly associated with carriage duration (Fig. 3). *M*. *haemolytica* carriage episodes were too few (prevalence 1.7–13.3%) to model using survival analysis but carriage density did extend transiently up to 6 log_10_ genome copies/ml. Given the infrequency of observed second episodes for both bacterial species, it was not possible to generate precise estimates for the median duration of carriage per episode type. Only two animals had sustained carriage of *H*. *somni* for the entire study, but twenty-six had sustained carriage of *P*. *multocida*. It would be of interest to define factors which determine persistence of carriage with these pathobionts.

A greater incidence of respiratory disease has been reported in male calves than in female calves^46^, suggesting sex may influence respiratory pathobiont colonisation dynamics. We included sex as a covariate in exponential survival models but did not find that it was significantly associated with carriage duration for either *H*. *somni* or *P*. *multocida*. Furthermore, when carriage rates observed at each occasion were stratified by sex there was no difference between the two proportions carrying on any occasion (data not presented). This may indicate that reported differences in disease rates may not be due to marked differences in carriage biology but instead to other factors^41^.

This study uses qPCR to quantify and track carriage of the target bacterial species over time in the nasal airways. A limitation of PCR is that it will detect DNA from inviable organisms that persist in the respiratory tract even when viable organisms are no longer present or cannot be detected by culture. It can be argued that specific detection of DNA from these unculturable organisms provides evidence of either current or recent colonisation and is thus of biological interest. In studies in humans we have often found samples positive by culture and negative by PCR^31^ perhaps reflecting that, on occasion, culture has the potential to be more sensitive than PCR when there are very small numbers of organisms present. Interestingly, we did not encounter this phenomenon in this study, perhaps reflecting the poor sensitivity of culture for these bacterial species. In common with all studies of mucosal colonisation, our study is subject to sampling error – we will have failed to detect colonisation on occasion by obtaining the swabs from the wrong place, at the wrong time or insufficiently frequently and, indeed, our choice of the nasal cavity may not have been optimal for detection for one or more of these organisms. Accordingly, our results are likely to be underestimates and there is a need to develop sufficiently non-invasive techniques which will permit more frequent or even continuous sampling with due consideration to animal welfare. More broadly, this is a study in healthy cattle, and we cannot be certain whether and to what extent the strains of bacteria they carry in the nose are associated with outbreaks of disease or how specific virulence factors may vary among the same bacterial species. Moreover, our failure to detect statistical significance, for instance in relation to possible associations among organisms, may be explained by the relatively small sample size.

For the three bacterial species studied, we observed unexpected marked differences in prevalence, carriage duration and density. These observed differences provide evidence for genuine differences in the carriage biology of these organisms and do not reflect sampling artefact. Quantitative molecular techniques have identified increased nasal bacterial carriage and density following respiratory viral infection in the upper respiratory tract of healthy mice and children^47,48^. We hypothesise density may be important for transmission of bovine pathobionts between herd members and may affect the likelihood of invasive disease. Moreover, respiratory viral infection may influence bacterial colonisation dynamics. The methodological approaches and carriage patterns presented in this report lay the foundation for well powered studies exploring transmission models of bovine respiratory disease.

## Materials and Methods

### Cattle and husbandry

Beef calves, either Charolais, Hereford or Limousin crosses, were bred at Rothamsted Research, North Wyke Farm, Devon, UK. In November 2015 at weaning, 60 calves aged 5 to 7 months were transferred to the Biotechnology and Biological Sciences Research Council (BBSRC) North Wyke Farm Platform National Capability^49^ and allocated to one of two identical, physically adjacent purpose-built cattle housing facilities, designated the ‘Green Barn’ and the ‘Red Barn’. Barns are highly standardised and representative of typical modern commercial winter housing facilities for beef cattle in the UK: solid concrete sidewalls to animal height (~1.5 m) and ventilated naturally above animal height with space boarding. Barns are orientated SW-NE such that the prevailing winds assist with their ventilation through end apertures (solid gates approximately 2 m high with openings above and a space-boarded gable end). Windbreaks closing the end apertures are used to provide further protection in adverse weather (strong winds, rain or low temperatures). Animals were allocated to housing, ensuring bodyweight, sire breed and sex were balanced between the two barns. In January 2016, within each barn, calves were separated into six pens each of five animals, nose-to-nose contact between animals within adjacent pens was permitted. Calves were housed on deep litter straw bedding with access to water and silage *ad libitum*. Animals were observed daily throughout all studies by animal technicians and any cattle showing abnormal behaviour that might have indicated poor health were examined in more detail by one of us (AT) using the Wisconsin scoring system for signs of respiratory disease (cough, nasal and ocular discharge, abnormal ear/head tilt and rectal temperature)^50^. However, no signs indicative of BRD or any other clinical problem were observed. Animals received no vaccines prior to or during the study, nor were antimicrobials administered to any animal during the course of the study.

### Sample collection

#### Nasal swabs

Nasal swabs were collected from each calf on five separate visits between January 2016–April 2016, on days: 0, 33, 47, 62 and 75 as follows: any excessive debris on nares was cleaned with a disposable paper towel. A swab 15 cm in length with breakable polyester tip (MW821 HydraFlock, Medical Wire & Equipment, Corsham, UK) was inserted to approximately 10 cm depth and rotated 360° against the mucous membranes, then withdrawn carefully, avoiding contact with other areas of the nasal cavity. The swab tip was aseptically broken off into 1.5 ml skim milk-tryptone-glucose-glycerol (STGG) medium. Swab samples were marked with a random number from 1–90 using pre-printed labels, maintained at 4 °C for no more than 3 hours and then vortexed to release bacteria into the STGG and frozen at −70 °C until further analysis^31,51^. Gloves were changed between handling each animal. On days 33 and 47 a dual-tipped polyester swab (MW821DC Dual Hydraflock, Medical Wire & Equipment, Corsham, UK) was collected; one tip was transferred to STGG and the other to RNAlater stabilisation solution (Thermo Fisher Scientific, UK) for gene expression studies (to be reported later). A nasal swab was not collected on day 47 from one animal (ID: 23 R) as difficulty was experienced collecting a sample from this individual.

#### Parasitological sampling

Shortly after housing in November 2015, composite samples of 10 freshly deposited faecal pats collected from the floor of each barn were tested for the presence of respiratory nematode larvae (*Dictyocaulus viviparus*) using the Baermann technique. Following parasitological testing, all calves were treated once routinely with 200 mg ivermectin (Noromectin®, Norbrook) pour-on (40 ml of product) to control respiratory and gastrointestinal nematodes and external parasites.

### Bacterial culture

*H*. *somni* American Type Culture Collection (ATCC) 43625, *M*. *haemolytica* ATCC 33396 and *P*. *multocida* ATCC 43137 were used as positive control strains for culture and PCR assay development. All reference strains used in the study (Supplementary Table S1) for PCR assay optimisation and specificity testing were cultured on Columbia blood agar supplemented with 5% sheep blood (CBA; Thermo Fisher Scientific, Basingstoke, UK) overnight at 37 °C with 5% CO_2_, except for *H*. *somni* and *Haemophilus influenzae*, which were cultured on Chocolate agar (E&O laboratories, UK) at 37 °C, for 24–48 hours with 5% CO_2_. All cultures were sub-cultured and Gram-stained to ensure purity.

For isolation of *Pasteurellaceae*, nasal swabs were thawed on ice, vortexed and a 50 µl STGG broth aliquot was spread over the entire surface of an agar plate^52^. For detection of *M*. *haemolytica* and *P*. *multocida* swabs were cultured on CBA, *Pasteurella* selective agar and MacConkey agar with salt (Thermo Fisher Scientific, Basingstoke, UK). *Haemophilus* selective agar (Thermo Fisher Scientific, Basingstoke, UK) and Chocolate agar plates (E&O laboratories, UK) were used for the enumeration of *H*. *somni*. Plates were cultured in an atmosphere containing 5% CO_2_ at 37 °C for 16–72 hours. Plates were examined after 16 hours and all colonies phenotypically resembling *M*. *haemolytica* or *P*. *multocida* were subcultured onto CBA, while presumptive *H*. *somni* colonies were subcultured onto Chocolate agar. Bacterial identification was done according to standard microbiological techniques for *H*. *somni*^53^, *M*. *haemolytica*^54^ and *P*. *multocida*^55^. For long term storage, bacterial cells were harvested into Brain Heart Infusion broth or *Haemophilus* Test Medium (*H*. *somni* only) supplemented with 20% glycerol (Media Services, School of Cellular and Molecular Medicine, University of Bristol, UK) and stored at −70 °C.

### DNA extraction

Frozen nasal swab samples and bacterial reference strains were thawed on ice and then vortexed. Each sample (300 µl) was aliquoted into a 2 ml extraction tube. Automated extraction of nucleic acid from samples was carried out in a QIAsymphony SP instrument (QIAGEN, CA, USA) using QIAsymphony DSP Virus/Pathogen Mini Kit (QIAGEN, CA, USA). An elution volume of 140 µl was produced from 200 µl of the 300 µl aliquot and stored at −70 °C. To determine successful DNA extraction and absence of PCR inhibition the bacteriophage T4 was used as an internal amplification control^31^.

### Real-time PCR for bacteria

Real-time TaqMan PCR (qPCR) assays targeting *sodA* for *M*. *haemolytica* and the 16S rRNA region of the bacterial genome for *H*. *somni* and *P*. *multocida* were performed, using previously published primers and TaqMan probes^32,56^. For *M*. *haemolytica* a novel TaqMan probe was designed to work with previously published primers (Table 4).

**Table 4:** Primer and probe (P/P) sequences used for qPCR assays in this study.

Target Species	Target	P/P Name	P/P Sequence (5′–3′)	Length	Melting Point (°C)	Reference
*M*. *haemolytica*	*sodA*	Mh-SGF	AGCAGCGACTACTCGTGTTGGTTCAG	26	65.6	Guenther *et al*.^32^
*M*. *haemolytica*	*sodA*	Mh-SGR	AAGACTAAAATCGGATAGCCTGAAACGCCTG	31	68.7	Guenther *et al*.^32^
*M*. *haemolytica*	*sodA*	Mh-BV1P*	TTCAACCGCTAACCAGGACAACCCAC	26	68.4	This study
*P*. *multocida*	16S rRNA	Pm-TMF	CGCAGGCAATGAATTCTCTTC	21	58.5	Mahony and Horwood^56^
*P*. *multocida*	16S rRNA	Pm-TMR	GGCGCTCTTCAGCTGTTTTT	20	58.3	Mahony and Horwood^56^
*P*. *multocida*	16S rRNA	Pm-TMP*	ACTGCACCAACAAATGCTTGCTGAGTTAGC	30	69.2	Mahony and Horwood^56^
*H*. *somni*	16S rRNA	Hs-TMF	AGGAAGGCGATTAGTTTAAGAGATTAATT	29	58.8	Mahony and Horwood^56^
*H*. *somni*	16S rRNA	Hs-TMR	TCACACCTCACTTAAGTCACCACCT	25	60.0	Mahony and Horwood^56^
*H*. *somni*	16S rRNA	Hs-TMP*	ATTGACGATAATCACAGAAGAAGCACCGGC	30	69.7	Mahony and Horwood^56^

*Probe fluorophore and quencher: 5′ FAM, 3′ BHQ-1.

Primers and probes were assessed and optimised using Primer Express Software v.3.0 (Life Technologies, USA), and specificity was assessed *in silico* by BLAST searches using the National Center for Biotechnology Information database (NCBI).

Extracts were thawed and centrifuged prior to PCR for 1 minute at 1000 × *g*. MicroAmp optical 384-well reaction plates (Life Technologies, USA) were prepared using a QIAgility pipetting robot and software (QIAGEN, CA, USA). The qPCRs were performed in a 20 µl volume consisting of 10 µl TaqMan® (Applied Biosystems), 5 µl Primer/Probe Mix (Sigma Aldrich) and 5 µl nucleic acid template. Working concentrations of primers and probe were 300 nM and 100 nM respectively. Cycling conditions on a ViiA7 real-time PCR instrument (Thermo Fisher Scientific) for *P*. *multocida*, *H*. *somni* and T4 (internal amplification control) assays were as follows: 95 °C for 20S hold stage, followed by 50 cycles of 95 °C for 3S and 60 °C for 60S. Cycling conditions on a QS7 real-time PCR instrument (Thermo Fisher Scientific) for *M*. *haemolytica* were employed as follows: 95 °C for 20S hold stage, followed by 50 cycles of 95 °C for 3S and 69 °C for 60S. Cross-reactivity was observed in this assay with *M*. *glucosida* CCUG 38457 T under the same cycling conditions used for *P*. *multocida*, *H*. *somni* and T4 as described above. A thermal gradient PCR was conducted and determined an alternative annealing temperature at 69 °C where no cross-reactivity was observed (data not presented). Fluorescence emission was measured at the end of the elongation step. No template and positive template controls were included in every run.

Real-time PCR data collected for *P*. *multocida*, *H*. *somni* and T4 were analysed using ViiA7 software (v1.1) and data collected for *M*. *haemolytica* were analysed using QuantStudio™ Real-Time PCR Software (v1.3). For all assays, auto baseline settings were employed, and threshold values manually set after all PCR runs were completed. Thresholds were set above any background amplification and approximately halfway through the exponential phase. Results were exported as csv files for further analysis.

### Specificity panel

To determine the specificity of the three assays, a panel of 40 bacterial strains (Supplementary Table S1) were selected based either on genetic relatedness to target organisms, known involvement with respiratory disease in ruminants, or being common bacteria of different genera. Specificity panel bacteria were cultured overnight as described above and diluted 10^−3^, 10^−4^, 10^−5^ in L6 lysis buffer (Public Health England, Bristol, UK), followed by automated DNA extraction using a QIAsymphony SP instrument (as described above).

### Growth curves in liquid broth culture

Log-phase liquid cultures of *H*. *somni*, *M*. *haemolytica* and *P*. *multocida* were used to construct a 10-fold dilution series for each organism, quantified at each dilution by culture and colony counting. Growth curves were performed in triplicate on separate days for *M*. *haemolytica* and *P*. *multocida* and in duplicate for *H*. *somni* (biological replicates). *M*. *haemolytica* and *P*. *multocida* reference strains were grown in liquid medium as described in^39,40^. In brief, 4–6 colonies from pure plate cultures were used to inoculate 20 ml Brain Heart Infusion (BHI) broth (Media Services, School of Cellular and Molecular Medicine, University of Bristol) and incubated overnight at 37 °C with shaking at 200 rpm. An aliquot of overnight inoculum was transferred to fresh BHI broth the next morning to achieve a starting optical density (OD) measured at 600 nm (Thermo Spectronic Genesys 6, Thermo Electron Scientific Instruments LLC, WI, USA) of 0.05 (approximately 10^7^ CFU/ml). After 1 hour, and at subsequent regular intervals 1 ml aliquots were taken for further OD measurements until stationary phase was reached. A final reading was taken at 24 hours. For *H*. *somni*, Veterinary Fastidious Medium (VFM) (Oxoid, Basingstoke, UK) was directly inoculated with 4–6 colonies from pure plate culture (starting OD of 0.05, approximately 10^7^ CFU/ml) and incubated at 37 °C, with shaking at 200 rpm and 5% CO_2_^57^. For all bacterial species, at late logarithmic phase, a 100 µl aliquot of liquid culture was taken and 10-fold serial dilutions prepared in 900 µl STGG. For *M*. *haemolytica* and *P*. *multocida* 50 µl aliquots of dilutions 10^−3^ to 10^−8^ of the series were plated out in triplicate onto BHI agar. For *H*. *somni*, 100 µl aliquots from dilutions 10^−3^ to 10^−8^ were plated onto Chocolate agar in triplicate. All plates were incubated for 12–24 hours at 37 °C, in 5% CO_2_ for colony counts. Counts over 750 were considered too numerous to count and where necessary were extrapolated from higher dilutions yielding lower counts. Bacterial counts were performed in triplicate and the mean expressed as log_10_ colony count/ml. After plating, the dilution series was immediately cooled and held frozen at −70 °C.

### Real-time PCR standard curves

Liquid broth cultures of each bacterial species were used to generate qPCR standard curves^31^ which were used to evaluate assay performance and to quantify template from bovine nasal swabs.

Dilutions of liquid cultures for each organism (described above) were thawed on ice and a 300 µl aliquot of each dilution (10^−1^ to 10^−10^) was inactivated at 100 °C for 10 minutes using a digital heat block (Grant Boekel, BBD, Grant Instruments, Cambridge, UK)^31^. Successful inactivation was confirmed through appropriate plate cultures for each species. Nucleic acid was extracted from all dilutions and PCR runs were conducted for each bacterial species as described above.

Standard curves were generated by linear regression of cycle quantification (C_q_) values versus log_10_ CFU/ml values for corresponding 10-fold serial dilutions of broth cultures. Five technical replicates of C_q_ values were performed at each dilution. The linear operating range, C_q_ cut-off value and amplification efficiency were determined for each assay. The amplification efficiency (*E*) was calculated based on the slope of the standard curve as follows: *E* (%) = (10^−slope^ − 1) × 100. The endpoint dilution of the standard curve at which tested samples were positive was used to determine C_q_ cut-off values.

### DNA sequencing

As culture confirmation proved technically difficult for this bacterial species, products generated from the *H*. *somni* PCR assay performed on nasal swabs were purified using a QIAquick PCR purification kit (Qiagen, CA, USA) according to the manufacturer’s instructions and sequenced (Eurofins Genomics, Ebersberg, Germany) with identification based on BLAST analysis of the target 16S rRNA gene.

### Standard curves

A linear regression model was fitted to colony counts and C_q_ values obtained from liquid culture as described above. The log_10_ mean colony count/ml was considered as the response variable. Mean C_q_ value (N = 5) was an explanatory variable. A parallel lines model was fitted to allow for differences in the intercept between biological replicates of the growth curves, obtaining the best estimate of the slope by assuming an additive effect of biological replicate. After fitting a parallel lines model, a predictive model was produced for each species using the mean intercept across the biological replicates. C_q_ values obtained from collected nasal swabs were converted to genome copies/ml by interpolation using the predictive models. *In vivo*, clinical samples are likely to contain both viable and non-viable bacterial cells. Accordingly, colony forming units/ml (CFU/ml) and genome copies/ml cannot be used interchangeably for these assays, and genome copies/ml are presented.

### Carriage and co-carriage

Rates of carriage of *H*. *somni*, *M*. *haemolytica* and *P*. *multocida* were calculated as the numbers of animals positive by qPCR for each agent divided by the numbers of animals sampled on each occasion, unless stated otherwise. Carriage estimates for each bacterial species were calculated for each barn (Green and Red) and as an overall average. Co-carriage rate, defined as carriage of either two or three of the bacterial species on the same occasion, was calculated as an overall average for the two barns. Confidence intervals for proportions and differences between proportions were calculated using the Wilson score^58^ and Newcombe-Wilson hybrid score^59^ methods respectively^60^. Levels of co-carriage of *H*. *somni*, *M*. *haemolytica* and *P*. *multocida* were assessed at each visit using Fisher’s exact test (because of expected values lower than 5) to assess for the independence of the carriage of pairs of different bacterial species, based on the counts of calves with different combinations of positive/negative qPCR results.

### Interval-censored survival analysis

Interval-censored survival analysis was used to estimate the rate (‘hazard’) of clearance of bacterial carriage. A parametric proportional hazards regression model for interval-censored data with an exponential distribution was fitted to carriage episodes from all animals (N = 60). An episode was defined as a period of uninterrupted *H*. *somni* or *P*. *multocida* carriage. Further details on rules defining carriage episodes and on dataset construction are provided in Supplementary Information, Method S2. The number of carriage episodes for each bacterium contributing to the survival analysis was not equal to the number of animals observed; some animals experienced multiple carriage episodes whilst others were carriage-free. Adjustment for correlation between recurrent episodes of carriage within the same animal was made by including animal identity as a clustering variable and calculating cluster robust standard errors^61^. The effects of sex and density of carriage when first positive were considered as covariates in univariable analyses. To assess the relationship between log hazard ratio and density (log_10_ genome copies/ml) we grouped the observations into categories based on the density quartiles. Parameter estimates for each density category were plotted to assess the fit. The hazard for each sex and density category was estimated and expressed as a hazard ratio relative to the baseline category. Median carriage duration was calculated as −log_e_(0.5) divided by the estimated hazard value. Improvement in model fit between the unconditional model (no covariates specified) and the model with the covariate of interest was assessed by calculating the log-likelihood test ratio statistic. The strength of the relationship between categorical covariates (sex/carriage density) and hazard of clearance was assessed using the hazard ratio p-value. All analyses were performed in R version 3.5.0, using base functions and the following supplementary packages: ggplot2 (version 2.2.1), longCatEDA (version 0.31^62^), icenReg (version 2.0.7^63^).

### Ethics statement

All animal studies were approved by the Rothamsted Research Ethical Review Board and animal care and use protocol protocols adhered to the Animals (Scientific Procedures) Act 1986 as revised 1 January 2013 to comply with European Directive 2010/63/EU, permit number (3003338).

## Supplementary information

Supplementary Information: Insights into Pasteurellaceae carriage dynamics in the nasal passages of healthy beef calves

## Data Availability

The datasets generated during and/or analysed during the current study are available from the corresponding author on reasonable request.
