# Author Correction: Insights into *Pasteurellaceae* carriage dynamics in the nasal passages of healthy beef calves

**DOI:** 10.1038/s41598-019-52010-1

**Published:** 2019-10-29

**Authors:** A. C. Thomas, M. Bailey, M. R. F. Lee, A. Mead, B. Morales-Aza, R. Reynolds, B. Vipond, A. Finn, M. C. Eisler

**Affiliations:** 1 Bristol Veterinary School, University of Bristol, Langford, UK; 2 Rothamsted Research, North Wyke, Devon, UK; 3 Rothamsted Research, Harpenden, UK; 4 Bristol Children’s Vaccine Centre, University of Bristol, Bristol, UK; 5 School of Cellular and Molecular Medicine, University of Bristol, Bristol, UK; 6 School of Population Health Sciences, University of Bristol, Bristol, UK; 7 Public Health Laboratory Bristol, Public Health England, Bristol, UK

Correction to: *Scientific Reports* 10.1038/s41598-019-48007-5, published online 16 August 2019

The Acknowledgements section in this Article is incomplete.

“The authors thank Rothamsted Research (BBS/E/C/000J0100) for hosting animal work; Hannah Fleming, Bruce Griffith and Simon White for excellent support and animal care during sample collection periods (Rothamsted Research); the late Robert Orr for initial contributions towards study conceptualisation and extensive knowledge of the North Wyke Farm Platform operations (Rothamsted Research); Debbie Langton for sequencing support (University of Bristol). The authors acknowledge the support from the NIHR Health Protection Research Unit in Evaluation of Interventions at University of Bristol.”

should read:

“This work was funded by the Biotechnology and Biological Sciences Research Council (BBSRC) under an Industrial Case Studentship (BB/J012483/1). The authors thank Rothamsted Research (BBS/E/C/000J0100) for hosting animal work; Hannah Fleming, Bruce Griffith and Simon White for excellent support and animal care during sample collection periods (Rothamsted Research); the late Robert Orr for initial contributions towards study conceptualisation and extensive knowledge of the North Wyke Farm Platform operations (Rothamsted Research); Debbie Langton for sequencing support (University of Bristol). The authors acknowledge the support from the NIHR Health Protection Research Unit in Evaluation of Interventions at University of Bristol.”

In addition, this Article contains ambiguous wording in the Results section, under subheading ‘Duration of carriage and hazard of clearance’. To clarify the findings, the following text should be disregarded:

“these trends were non-linear with categories 2 and 4 associated with a longer duration (lower hazard) compared to category 3, and category 4 (highest density) associated with the longest subsequent carriage duration (Table 3).”

