# Supplementary material for: Insights into Pasteurellaceae carriage dynamics in the nasal passages of healthy beef calves
Source: Sci Rep. 2019 Aug 16;9:11943. doi: 10.1038/s41598-019-48007-5 (PMC6697682; doi:10.1038/s41598-019-48007-5)
Supplement: Supplementary file 1 — Supplementary Information: Insights into Pasteurellaceae carriage dynamics in the nasal passages of healthy beef calves [file 41598_2019_48007_MOESM1_ESM.docx]

**Supplementary Information: Insights into *Pasteurellaceae* carriage dynamics in the nasal passages of healthy beef calves**

A.C. Thomas*^1,2,4^, M. Bailey^1^, M.R.F. Lee^1,2^, A. Mead^3^, B. Morales-Aza^4,5^,
R. Reynolds^6^, B. Vipond^7^, A. Finn^¶4,5,6^, M.C. Eisler*^¶1^

^1^Bristol Veterinary School, University of Bristol, Langford, UK 
^2^ Rothamsted Research, North Wyke, Devon, UK
^3^ Rothamsted Research, Harpenden, UK
^4^Bristol Children’s Vaccine Centre, University of Bristol, Bristol, UK ^5^School of Cellular and Molecular Medicine, University of Bristol, Bristol, UK
^6^School of Population Health Sciences, University of Bristol, Bristol, UK ^7^Public Health Laboratory Bristol, Public Health England, Bristol, UK

* Corresponding author

E-mail: amyc.thomas@bristol.ac.uk; mark.eisler@bristol.ac.uk

^¶^ These authors contributed equally to this work.

**Table S1: Bacterial strains used in this study**

| **Species** | **Strain/reference designation** | **Source** |
| --- | --- | --- |
| *Actinobacillus equuli* | NCTC* 8529 | Equine, blood |
| *Actinobacillus ligieresii* | NCTC 4189 | Bovine, sub-maxillary gland |
| *Actinobacillus pleuropneumoniae* | NCTC 11383 | Ovine, arthritis |
| *Actinobacillus suis* | APHA^†^ IS14-13758 | Species unknown, liver |
| *Bibersteinia trehalosi* | APHA IS21-03264 | Bovine, location unknown |
| *Enterococcus faecalis* | JH2-2 | Unknown |
| *Escherichia coli* | ATCC®^‡^ 25922 | Species unknown, clinical isolate |
| *Fusobacterium necrophorum* subsp. *necrophorum* | APHA C977 | Species unknown, foot |
| *Haemophilus parainfluenzae* | UoB^§^, from other carriage studies | Unknown |
| *Histophilus somni* | ATCC® 43625, type strain | Bovine, brain |
| *Mannheimia glucosida* | CCUG^¥^ 38459 | Ovine, lung |
| (n = 4) | CCUG 38460 | Ovine, lung |
|  | CCUG 38467 | Ovine, lung |
|  | CCUG 38457, type strain | Ovine, lung |
| *Mannheimia granulomatis* | CCUG 45422, type strain | Bovine, subcutaneous granuloma |
| *Mannheimia haemolytica* | ATCC® 33396, type strain | Ovine, location unknown |
| (n = 4) | UoB, V1-53-2 | Bovine, nasal swab |
|  | UoB, V1-71-1-(19) | Bovine, nasal swab |
|  | UoB, V1-37-1 | Bovine, nasal swab |
| *Mannheimia ruminalis* | CCUG 38470, type strain | Ovine, rumen |
| *Mannheimia varigena* | APHA IS12-04533 | Bovine, lung |
| **Table S1 continued** |  |  |
| *Moraxella bovis* | NCTC 11013 | Bovine, eye |
| *Moraxella bovoculi* | UoB, V1-26-1 | Bovine, nasal swab |
| Moraxella catarrhalis | ATCC® 25240 | Unknown |
| *Mycobacterium bovis* | BCG, Glaxo | Unknown |
| *Mycoplasma bovis* | ATCC® 25523, type strain | Bovine, mastitis |
| *Pasteurella canis biotype 2* | NCTC 11621, type strain | Canine, throat |
| *Pasteurella multocida* | ATCC® 43137, type strain | Porcine, location unknown |
| (n=4) | UoB, V1-38-5 | Bovine, nasal swab |
|  | UoB, V1-52-2 | Bovine, nasal swab |
|  | UoB, V1-41-1 | Bovine, nasal swab |
| *Pseudomonas aeruginosa* | UoB, from other carriage studies | Unknown |
| *Rhodococcus equi* | NCTC 10673 | Unknown |
| *Salmonella dublin* | APHA R07571 | Bovine, lung |
| *Salmonella typhimurium* | DT104 strain 30 | Bovine, faeces |
| *Staphylococcus aureus* | ATCC® 25923 | Clinical isolate |
| *Streptococcus agalactiae* | ATCC® 12403, type strain | Fatal septicaemia |
| *Streptococcus pluranimalium* | APHA C06551 | Bovine, nasal swab |
| *Streptococcus suis* | APHA 25 | Bovine, lung |
| *Truepella pyogenes* | NCTC 5224 | Porcine |
| * NCTC: National Collection of Type Cultures; ^†^ APHA: Animal and Plant Health Agency;  ^‡^ ATCC®: American Type Culture Collection; ^§^ UoB: University of Bristol;  ^¥^ Culture Collection University of Gothenburg | | |

**
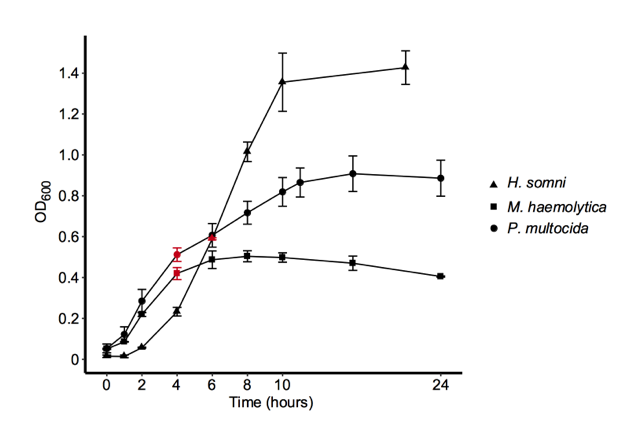
**

**Figure S1: Liquid broth culture of *Histophilus somni*, *Mannheimia haemolytica* and *Pasteurella multocida*.** Optical density at 600 nm (OD_600_) of the cultures of the three species was measured approximately every 2 hours up to 24 hours. Data for time points at which cells were harvested at log phase for generation of qPCR standard curves are shown in red. The results are the mean OD from two independent experiments (*H. somni*) and three independent experiments (*M. haemolytica* and *P. multocida*). Error bars represent the standard error of the mean.

**
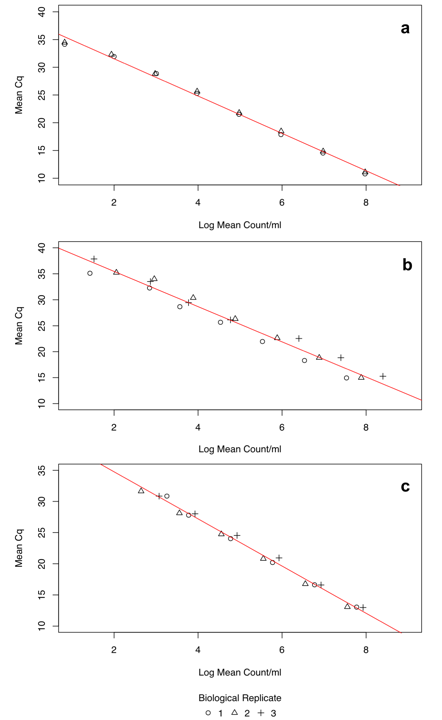
**

**Figure S2: Standard curves used for conversion of C_q_ values into genome copies/ml.** *Histophilus somni* (panel a) liquid growth performed in duplicate on two different days. *Mannheimia haemolytica* (panel b) and *Pasteurella multocida* (panel c) liquid growth performed in triplicate on three different days. Mean C_q_ values obtained from 5 technical PCR replicates at each dilution, for each biological replicate. Each biological replicate is represented by a symbol: circle, triangle or cross. Colony counts are expressed as the mean of three repeats.

**Table S2: Presence of Histophilus somni, Mannheimia haemolytica and Pasteurella multocida determined by culture and qPCR on bovine nasal swabs (N = 60) collected on day 0 from healthy cattle.**

| **Species** | **qPCR** | **Culture** | **Total** |
| --- | --- | --- | --- |
| *H. somni* | + | + | 1 |
|  | - | + | 0 |
|  | + | - | 28 |
|  | - | - | 31 |
| *M. haemolytica* | + | + | 4 |
|  | - | + | 0 |
|  | + | - | 5 |
|  | - | - | 51 |
| *P. multocida* | + | + | 43 |
|  | - | + | 0 |
|  | + | - | 14 |
|  | - | - | 3 |

**Method and data S1: Effect of pen and barn on bacterial carriage rates**

Differences in the carriage of *Histophilus somni* and *Pasteurella multocida* between animals in pens within two barns was investigated prior to combination of animals into one cohort (N = 60) for survival analyses*.* For both bacterial species, datasets were constructed by counting the number of calves positive and negative for carriage (response) for each pen within each barn. General linear models with a Poisson error distribution and log-link function were fitted using the base ‘glm’ function of R version 3.5.0.

A sequence of nested multi-level models were built from the baseline model (M0); response of carriage plus main effect of barn and nested effect of pen within barn. Two additional models were considered: M1 = baseline (M0) plus the effect of barn on carriage; and M2 = model M1, plus the effect of pen nested within barn on carriage. The impact of additional terms included in a more complex model was assessed by comparing the change in residual deviance from a nested, simpler model with the upper-tailed critical value (*p* < 0.05) of the appropriate chi-squared distribution (as identified by the change in the residual degrees of freedom between the models).

There was no evidence for differences in carriage rates between barns for *P. multocida* on any sampling occasion (day 0, p = 0.549; day 33, p = 0.487;
day 47, p = 0.824; day 62, p = 0.0617; day 75, p = 0.174). No evidence for barn differences were found for *H. somni* on day 0 (p = 0.0693) or day 47 (p = 0.0979), but evidence was found on day 33 (p = 0.001), day 62 (p < 0.001) and day 75
(p < 0.001). Differences in carriage rates between pens were found on two occasions only: *H. somni* on day 47 (p = 0.0178) and *P. multocida* on day 62
(p = 0.0199).

**Method S2: Interval-censored survival analysis**

**Rules to define carriage episodes**

Interval-censored survival analysis was used to estimate the rate (‘hazard’) of clearance of bacterial carriage and the median duration of carriage. Models were fitted in R (version 3.5.0) using the package icenReg^[[1]](#footnote-1)^.

The following definitions, rules and assumptions were applied:

1. Carriage episode: a period where a calf is positive for *H. somni* or *P. multocida*. A period may span multiple visits/observations if carriage is detected consecutively without interruption. If carriage is interrupted, i.e. lost and then re-acquired, the period following re-acquisition of carriage constitutes a new carriage episode.
2. The covariates, sex and density when first positive, were assumed to be time-invariant.
3. The maximum possible duration of carriage was from the first carriage-positive visit to the day before the first following carriage-free visit.
4. The minimum possible duration of carriage was set from the first to the last positive visit in the series, assuming carriage was uninterrupted between visits.
5. If the episode was interrupted by one or more missed visit’s then the minimum duration was up to the last positive visit *before* the first missed one.
6. If the last positive visit was the calves last visit in the study, then the duration was right-censored at 365 days (otherwise it would have been infinite).

Follow-up was interrupted by one missed visit for one calf (ID 23, day 47). This missed visit interrupted a carriage-free episode and therefore it was not necessary to define a carry-free interval. Recurrent-episodes of carriage and non-carriage were distinguished for each calf when observed, for example episode 1, 2 and 3. No more than 3 episodes could be experienced by one calf. Carriage rates for *M. haemolytica* were too low to model.

**Dataset** **construction**

Datasets were constructed prior to analysis in R. Briefly, minimum and maximum carriage intervals were defined for each possible carriage permutation using the rules outlined above and inputted into a VLOOKUP table in Microsoft® Excel. There were 32 different carriage scenarios possible.

Carriage or no carriage for each animal at each visit was coded as either 1 or 0 respectively. This resulted in each animal having a five-digit binary number representing their carriage on each visit of the study. Similarly, all possible carriage permutations (N = 32) were coded as a five-digit binary number, with each binary number corresponding to its decimal number (from 0 to 31); creating a code. This decimal code was inputted into the same VLOOKUP table and corresponded to a defined carriage interval, allowing future lookup of any given carriage permutation from an animal.

1. Anderson-Bergman C. icenReg: Regression Models for Interval Censored Data in R. Journal of Statistical Software. 2017;81(12):1-23. [↑](#footnote-ref-1)
